# Pharmaceutical Care in Primary Healthcare—A Study of Nurses’, Pharmacists’, and Physicians’ Experiences of Interprofessional Collaboration

**DOI:** 10.3390/healthcare12111100

**Published:** 2024-05-27

**Authors:** Ann Karin Helgesen, Carina Marie Nome, Julie Kjølhede Stenbjerg, Marie Helen Arnesen, Tina Reinertsen Aardalen, Tinne Dilles, Vigdis Abrahamsen Grøndahl

**Affiliations:** 1 Faculty of Health, Welfare, and Organisation, Østfold University College, P.O. Box 700, NO-1757 Halden, Østfold, Norway; vigdis.a.grondahl@hiof.no; 2 Haugvoll Nursing Home, Sarpsborg Kommune, Myrvollveien 25, NO-1743 Klavestadhaugen, Sarpsborg, Norway; 3 Emergency Room, Fredrikstad Kommune, Jens Wilhelmsens Gate 1, NO-1671 Kråkerøy, Fredrikstad, Norway; 4 Home Nursing Care Centre, Fredrikstad Kommune, Faunsvei 3-6, NO-1654 Sellebakk, Fredrikstad, Norway; 5 Intensive Care Unit, Østfold Hospital Trust, Kalnesveien 300, NO-1714 Grålum, Sarpsborg, Norway; 6 Centre for Research and Innovation in Care, Nurse and Pharmaceutical Care, University of Antwerp, Prinsstraat 13, 2000 Antwerp, Belgium; tinne.dilles@uantwerpen.be

**Keywords:** home nursing care, nursing home, interprofessional collaboration, nurses’ role, pharmaceutical care, primary healthcare, qualitative study

## Abstract

Implementation of pharmaceutical care for the benefit of patients and health services has been highlighted worldwide. Interprofessional collaboration between nurses, pharmacists, and physicians may contribute to raising awareness of pharmacological challenges, increasing quality, and minimising errors in pharmaceutical care. This study aimed to investigate how nurses, pharmacists, and physicians experience interprofessional collaboration in pharmaceutical care within community healthcare in Norway. The study had an explorative and descriptive design with a qualitative approach. Individual interviews were conducted with 12 healthcare personnel with key roles in pharmaceutical care within community healthcare services. The data were analysed using systematic text condensation. The results revealed three categories and four subcategories: The category “Professional challenges” contained the subcategories “Blurred responsibilities” and “The importance of trust and continuity”. The category “Organisational barriers” contained the subcategories “Lack of information exchange and suitable communications channels” and “Lack of time and meeting places”. The third category was “Nurses—the important link”. This study reveals challenges to and factors of success in increasing high-quality and safe pharmaceutical care, knowledge that can be used in quality work in the community health services and as input in curriculum development for the three professions.

## 1. Background

In recent years, implementation of pharmaceutical care for the benefit of patients and health services has been highlighted worldwide [1]. In 1990, pharmaceutical care was defined by Hepler and Strand as “the responsible provision of drug therapy for the purpose of achieving definite outcomes that improve a patient’s quality of life”, which “involves the process through which a pharmacist co-operates with a patient and other professionals in designing, implementing and monitoring a therapeutic plan that will produce specific therapeutic outcomes for the patient” [2]. More than twenty years later, the board of the Pharmaceutical Care Network Europe (PCNE) redefined the concept to “Pharmaceutical Care is the pharmacist’s contribution to the care of individuals in order to optimize medicine use and improve health outcomes” [3]. These two definitions by Hepler and Strand and the PCNE have in common that they emphasise the pharmacists’ role only. Nevertheless, pharmaceutical care does not stand alone but must be embedded in multidisciplinary treatment. It is well recognized that there is a need for interprofessional collaboration in pharmaceutical care to improve care quality and patient outcomes [4]. In 2023, the concept of pharmaceutical care still lacked consensus but was broadly discussed [5]. In this study, pharmaceutical care is defined as “Healthcare professionals’ contribution to the care of the individual in order to optimize medicine use and improve health outcomes” [6].

Due to an ageing population and the increase in long-term conditions, the number of people with multiple health conditions is set to rise. Most of these people are dependent on primary care services, which are described as the heart of integrated people-centred healthcare in many countries [7]. People with multimorbidity are at higher risk of safety issues such as polypharmacy, which may lead to poor medication adherence and adverse drug events. Given that guidelines currently in use are based on treatment of a single diagnosis, it is challenging to treat this patient group [8,9]. This put demands on interprofessional collaboration, as these patients will probably need help from all parts of the health service. Previous research has illuminated the fact that interprofessional collaboration between nurses, pharmacists, and physicians makes it easier to detect drug-related problems and helps to reduce inappropriate drug treatment [4,10,11].

An ever-ageing population and multimorbid patient groups exposed to polypharmacy constitute a challenge in primary healthcare. According to research, interprofessional collaboration between nurses, pharmacists, and physicians may contribute to raising awareness of pharmacological issues, increasing quality, and minimising errors in pharmaceutical care [4,6,11]. The nurse’s role in interdisciplinary pharmaceutical care is not transparent and varies between European countries. Similarly, in curricula for nurse education, the content of pharmaceutical care varies a lot. With this background, a European project entitled “Development of a model for nurse’s role in interprofessional pharmaceutical care” was carried out (Erasmus+: DeMoPhac (2018-1-BE02-KA203-046861). The present study, whose objective is to gain more knowledge about interprofessional collaboration in pharmaceutical care within community healthcare, is part of this European project and focuses on the Norwegian results.

The aim of this study was to investigate how nurses, pharmacists, and physicians experience interprofessional collaboration in pharmaceutical care within community healthcare in Norway.

## 2. Materials and Methods

### 2.1. Design

The study had an explorative and descriptive design with a qualitative approach [12]. The COREQ (COnsolidated criteria for REporting Qualitative research) checklist was used [13].

### 2.2. Setting and Participants

The study was conducted in home nursing care and nursing homes in the southeast of Norway during 2019.

For inclusion, participants had to be working as a nurse, physician, or pharmacist in the context of home nursing care or nursing homes and play a key role in pharmaceutical care. In total, 20 healthcare personnel who had a key role in pharmaceutical care were contacted by telephone or email and informed about the study by one of the authors. Those who were interested were asked their permission to send them further information about the study and a written request for participation. The final sample consisted of 12 participants: four nurses, four physicians, and four pharmacists. Two representatives from each profession participated from each respective scenario.

### 2.3. Data Collection

We used a semi-structured interview guide comprising topics concerning interprofessional collaboration in pharmaceutical care as well as the strengths, weaknesses, opportunities, and threats regarding the nurses’ role in interprofessional pharmaceutical care. Four of the authors (C.M.N., J.K.S., M.H.A. and T.R.A.) carried out the individual interviews in pairs; one performed the interview, and the other observed and tracked the time. The interviews were conducted at the participants’ workplace or the university college, according to the participants’ wishes. All interviews were carried out without interruption and were recorded and transcribed verbatim. The interviews lasted between 24 and 60 min (mean 45 min).

### 2.4. Data Analysis

The data were analysed using Malterud’s (2018) systematic text condensation [14]. In the first step, we defined preliminary topics by reading through all the transcribed interviews. The transcribed interviews were read several times by all the authors. After some discussion within the research team, the preliminary topics were identified. The next step was to organise and sort the data into code groups using colours and number codes. Meaningful units were organised into code groups. In step three, the code groups were sorted into subcategories. Then, a condensate was made based on the content of the meaningful units. In the final step, the pieces were put together again by using the condensate to make an analytic text for each subcategory. To validate the results, the transcripts were read by all the authors to check that there was consensus between the analytical text and what was originally said.

## 3. Results

Three categories, with a total of four subcategories, were developed from the data describing nurses’, physicians’, and pharmacists’ experiences with interprofessional collaboration in pharmaceutical care within community healthcare. The category “Professional challenges” contained the subcategories “Blurred responsibilities” and “The importance of trust and continuity”. The category “Organisational barriers” contained the subcategories “Lack of information exchange and suitable communications channels” and “Lack of time and meeting places”. The third category was named “Nurses—the important link” (see Table 1).

### 3.1. Professional Challenges

Participants from the three professions described challenges due to blurred responsibilities in pharmaceutical care. Unclear roles and a lack of clarification of responsibilities were challenging in interprofessional collaboration. Collaboration was dependent on trust in each other and continuity.

#### 3.1.1. Blurred Responsibilities

All participants experienced frequent overlap in the different professions’ roles within interprofessional collaboration. They stated that their responsibilities appeared rather unclear and random. A pharmacist related to home nursing care stated:

*“In an interprofessional situation with a patient, the patient’s clinical condition will clearly be the nurse’s responsibility. But with a physician present, I’m unsure how it is distributed.”*

Several participants also expressed uncertainty about others’ expectations of them and the collaborating professions. A nurse working in a nursing home called for regular collaboration meetings where the expectations of the various roles could be clarified:

*“Regular collaboration meetings will mean that you are aware of what they expect from us, what we expect from them. The work they do and the work we do.”*

Physicians working in home nursing care described that they did not have sufficient knowledge of the nurses’ tasks and competence. A pharmacist working in a nursing home had a similar experience and described it as follows:

*“I think maybe some of the weaknesses are that we may not have full insight into what the nurses or physicians or pharmacist can do. What is the difference between those professions in relation to what responsibility and knowledge/skills you have.”*

#### 3.1.2. The Importance of Trust and Continuity

All participants stated that collaboration became easier when they knew and trusted each other. One physician in a nursing home described it as follows:

*“The collaboration grows when you know each other, and one trusts more in each other eventually.”*

Another physician working in a nursing home highlighted the fact that interprofessional collaboration between nurses, physicians, and pharmacists can produce synergy. He described the collaboration as:

*“That all three together accomplish more, than each of them by themselves”.*

In home nursing care, physicians stated that they trusted the nurses’ observations and respected the nurses’ special relationship with the patients. The participants from nursing homes pointed out the importance of continuity in the collaboration through full-time positions. The advantage of having the same person to collaborate with over time was highlighted, as it was easier to get in touch with this person if they needed help. Some nurses and pharmacists had the impression that physicians lacked confidence in them. A nurse in a nursing home expressed the feeling of being overruled by a physician about an observation she made:

*“I want the physician to trust our observations more, they often overrule us in our observations. I wish they had a little more respect for the job we do.”*

### 3.2. Organisational Barriers

Participants described various organisational barriers to interprofessional communication. There was a lack of sufficient information exchange and suitable communication channels, as well as time and meeting places.

#### 3.2.1. Lack of Information Exchange and Suitable Communication Channels

All three professions described that communication in interprofessional collaboration was essential. It was agreed that mutual information exchange was crucial to providing optimal pharmaceutical care for the patients; however, in reality, this was lacking. Physicians related to home nursing care experienced limited information exchange between the three professions. They experienced it as a demand to be solely responsible for pharmaceutical care and felt that in some cases, they lacked sufficient information from the nurses. A physician in home nursing care described it as follows:

*“Physicians can find it difficult to discontinue medication when the patient has been on it for a long time, and they (the physician) are solely responsible for it. Physicians are responsible, but they sometimes lack the information that the nurses have at the time.”*

All participants from home nursing care experienced a scarcity in the exchange of information about the patients’ treatment with the pharmacist. The pharmacists had a more advisory function. The nurses experienced some collaboration with pharmacists, but this presupposed that the nurses took the initiative. A nurse in a nursing home explained:

*“It often happens that the nurse must consult a pharmacist. There is no pharmacist who calls us and asks. And I do not know if we should expect that. No, I do not think so.”*

A nurse working in home nursing care described that she was afraid of being perceived as insecure in her nursing role if she contacted a pharmacist for professional advice:

*“Should I contact a pharmacist and ask for advice now, or will it be perceived as if I’m more insecure in my role as a nurse?”*

Several physicians in the study emphasised the importance of good written documentation and record keeping from the nurses; at the same time, the physicians were aware that they had to be clear about what observations and feedback they actually wanted. Nurses in nursing homes found that physicians often provided verbal information, which could easily be misinterpreted:

*“Physicians provide information and instructions to nurses. Often verbally, and not always in writing. It can quickly become a misinterpreted situation.”*

A lack of communication channels between the various health services was mentioned by the participants from home nursing care. Nurses and physicians said that the electronic messaging systems between primary and specialist healthcare services did not work in an optimal way. The nurses received insufficient information from the hospital concerning changes in the patient’s medication lists. A physician described it as follows:

*“The nurse spends a lot of time calling the hospital, calling around and the physician also spends a lot of time finding the right medication lists, so it’s a big weakness today.”*

#### 3.2.2. Lack of Time and Meeting Places

Most participants stated that limited financial resources and lack of facilitation for interprofessional working made it difficult to collaborate. They had too little time for interprofessional collaboration in pharmaceutical care. Many emphasised that the nurses in particular had too many tasks and too little time for patients and for collaboration. A physician associated with a nursing home stated:

*“The nurses have too little time for each patient, and we have too little time to collaborate interprofessionally, it is somehow not appropriate.”*

In home nursing care, physicians stated that a lack of time and available meeting places made it difficult to conduct collaborative meetings:

*“We could have had collaboration meetings, but I don’t think it will be easy to carry out that you meet more often. I think it will be an annoyance if you must spend too much time for such meetings when we don’t get anything out of it. Neither me nor the nurses have time for that.”*

A physician in a nursing home described that collaborative meetings had to be structured and planned; otherwise, they quickly lost direction. Several nurses stated that collaboration was challenging when not all professions were physically present in the same health service. This could hinder opportunities for continuity. A pharmacist associated with a nursing home stated:

*“It is very difficult… if you are not in the same house and you are far apart from each other.”*

A nurse in a nursing home said that she did not contact the pharmacist, since she considered it unnatural to cooperate with pharmacists when they were not located in the same building:

*“I think if you had worked in the same place as pharmacists, it might have been more natural to collaborate. Here at the nursing home, we don’t have pharmacists and it is not natural for me to report to a pharmacist that I’ve never met, just talked to on the phone.”*

### 3.3. Nurses—The Important Link

All the participants said that the nurses had a central role in communication between the various professions, due to the nurses’ knowledge of the patient. The three professions stated that they were dependent on the nurses’ observations and knowledge of the patients. A pharmacist from a nursing home described it this way:

*“The nurses have a greater overview and see the challenges around the patient, because they know the patient well.”*

All the participants associated with home nursing care said that the nurses were the link in communication between the physician, the pharmacist, and the patient. One pharmacist said:

*“The nurse often becomes the key person or in a way the facilitator of the various issues on which patients depend.”*

Nurses in home nursing care described a greater amount of responsibility within pharmaceutical care than did the nurses in nursing homes. They explained that the physicians were more often present in the nursing homes and were more available for collaboration. The nurses described themselves as the link in pharmaceutical care, but felt that they had too much responsibility without having sufficient pharmaceutical competence:

*“I think we consider the effects and side effects of drugs too little. We must also discuss whether the effect outweighs the side effect of the drug. Weigh the pros and cons. I think we do that too little.”*

A physician associated with a nursing home considered that his own profession played the key role, but said that there was a need for all three professions within pharmaceutical care:

*“I think what strikes me is that the pharmacists have a good theoretical knowledge of medicine. Nurses have a very practical knowledge of the patient and how he feels, and what he takes and how he takes it, and the physician is a kind of middle ground between them.”*

## 4. Discussion

The results showed that nurses, physicians, and pharmacists experienced the interprofessional collaboration in pharmaceutical care within community healthcare as professionally challenging and felt that there were organisational barriers to collaboration. They also revealed that the nurse was an important link in collaboration.

A previous scoping review identified seven responsibilities and tasks of nurses in pharmaceutical care across various healthcare settings from all continents. The responsibilities included management of therapeutic and adverse effects of medication, management of medication adherence, management of patient self-medication, management of patient education and information about medication, prescription management, medication safety management, and transition of care coordination [15]. These responsibilities may vary between the individual nurses and between countries due to differences such as educational level and legal and legislative differences [6]. One study found that nurses became willing to be more involved in monitoring possible adverse drug reactions with increasing competence [16]. Furthermore, the quality of interprofessional collaboration in medication was found to be influenced by how much knowledge each professional had of the others, how they valued the others’ particular skills and expertise, and to what degree they respected each person’s unique contribution to the collaboration [16,17]. This is in line with the results of our study, whereby the importance of trust and continuity was one of the professional challenges when nurses, physicians, and pharmacists were collaborating in community healthcare services.

Nursing competence, periodic interprofessional consultation, and ad hoc interprofessional communication are identified as requirements for nurses to be able to collaborate with physicians and pharmacists in home nursing care settings. Collaboration between these professions was found to be suboptimal, and reservations about the nurses’ competencies were expressed [16]. When the competence of the nurse was questioned, questions were also raised regarding whether the nurses’ role was clearly described. Guidelines describing the role of nurses were mentioned as a requirement [10], which highlights the finding in our study wherein the responsibility of the different stakeholders was experienced as blurred. A Delphi study was recently undertaken with experts in order to reach an agreement on nurses’ competences for tasks in interprofessional pharmaceutical care. The experts agreed on 60 competences for 22 nursing tasks within pharmaceutical care [18].

A lack of information exchange and suitable communication channels were found to be organisational barriers in our study. Periodic interprofessional meetings for discussing pharmaceutical issues have been shown to improve the quality of pharmaceutical care for patients in home nursing care settings [19]. Lack of time was found to be another organisational barrier in our study, as it was in other studies [6].

The nurses, physicians, and pharmacists in our study said that nurses were important links and coordinators in pharmaceutical care in community healthcare services. The role of nurses in home nursing care is described as observing, recognising, and communicating information for regular clinical medication reviews to physicians that can prescribe and to community pharmacists, as well as informing and educating patients [10]. This is quite natural, because home nursing care nurses are the only healthcare professionals visiting the patients at home on a regular basis [10,20]. Previous studies indicate that interprofessional collaboration in clinical practice between nurses, physicians, and pharmacists might improve patient safety and quality of pharmaceutical care [11,21,22,23,24,25,26].

### Strengths and Limitations

A total of 12 participants took part in the study, which might seem like a small number; however, they all had a key role in pharmaceutical care in community health services [27,28]. Six participants were connected to nursing homes, and six to home nursing care. The interviews were conducted by pairs of master’s students, and a total of four master’s students were responsible for conducting all the interviews. The master’s students are not experienced research interviewers, but all four have worked as nurses for several years and have broad experience in interviewing patients and relatives [29]. In addition, the master’s students participated in an intensive training programme regarding pharmaceutical care and the use of qualitative methodology. To increase a joint understanding of the interview guide, the guide was discussed in the research group before the interviews took place [30]. The participants chose where they wanted to be interviewed, so that they felt comfortable [31]. The mean time for interviews was 45 min, and the interviews were rich in data. The six authors discussed the content and labelling of the categories and subcategories until consensus was reached, to ensure that these were in line with the content of the interview and the aim of the study [14]. The COREQ checklist was applied to ensure explicit and comprehensive reporting of our qualitative study [13].

## 5. Conclusions

Nurses, physicians, and pharmacists all experienced challenges due to blurred responsibilities, unclear roles, and lack of continuity in collaboration within pharmaceutical care. Lack of suitable communication channels, meeting places, and time for information exchange were other barriers to collaboration. The nurse was described by all three stakeholders as having a central role in the collaboration between the professions due to the nurses’ knowledge of the patient. The results reveal the need to set up regular interprofessional team meetings in primary healthcare. Digital tools can facilitate interprofessional alignment in an efficient way. Frameworks on healthcare providers’ roles and responsibilities concerning pharmaceutical care might stimulate discussions that can improve interprofessional collaboration in order to avoid misunderstandings between the various professionals.

### Implications for Practice

By asking key stakeholders (nurses, pharmacists, and physicians) in community health services how they experience interprofessional collaboration in pharmaceutical care, challenges to and factors of success in increasing high-quality and safe pharmaceutical care have been illuminated. This knowledge can be used both in quality work in the community health services and as input in curriculum development for the three professions.

## Figures and Tables

**Table 1:** Overview of the categories and the subcategories.

Professional Challenges	Organisational Barriers	Nurses-the Important Link
Blurred responsibilities	Lack of information exchange and suitable communication channels	
The importance of trust and continuity	Lack of time and meeting places	

## Data Availability

The qualitative data are not publicly available due to the consent the participants gave but are available from the corresponding author on reasonable request.
